# Myeloid Derived Suppressor Cells in Chronic Myeloid Leukemia

**DOI:** 10.3389/fonc.2015.00107

**Published:** 2015-05-15

**Authors:** Cesarina Giallongo, Nunziatina Parrinello, Maria Violetta Brundo, Salvatore Antonino Raccuia, Michelino Di Rosa, Piera La Cava, Daniele Tibullo

**Affiliations:** ^1^Division of Haematology, AOU “Policlinico-Vittorio Emanuele”, University of Catania, Catania, Italy; ^2^Department of Biological, Geological and Environmental Sciences, University of Catania, Catania, Italy; ^3^Institute for Agricultural and Forest Systems in the Mediterranean, National Research Council, Catania, Italy; ^4^Department of Biomedical and Biotechnology Sciences, University of Catania, Catania, Italy

**Keywords:** myeloid derived suppressor cells, chronic myeloid leukemia, Gr-MDSCs, PMNs

## Abstract

The suppression of the immune system creates a permissive environment for development and progression of cancer. One population of immunosuppressive cells that have become the focus of intense study is myeloid derived suppressor cells (MDSCs), immature myeloid cells able to induce immune-escape, angiogenesis, and tumor progression. Two different subpopulations have been identified and studied: granulocytic and monocytic MDSCs, with a different immunophenotype and immunosuppressive properties. Recently, an accumulation of both Gr-MDSCs and Mo-MDSCs cells has been found in the peripheral blood of chronic myeloid leukemia (CML) patients. They are part of the tumor clone showing BCR/ABL expression. Imatinib therapy decreases both MDSCs and arginase 1 levels to normal ones. This review will focus on actual knowledge for human MDSCs and their immunosuppressive activity in CML patients, with a critical attention to comparison of Gr-MDSCs and polymorphonuclear cells (PMNs). We will then suggest the monitoring of MDSCs in patients who have discontinued tyrosine kinase inhibitors (TKIs) therapy to evaluate if their increase could correlate with disease relapse.

## Introduction

The research on myeloid derived suppressor cells (MDSCs) has become the focus of intense study in recent years. For many years, the studies on MDSCs in cancer have been driven by murine experiments where they are described as CD11b/Gr-1 double-positive cells (1). The Gr-1 antigens Ly-6G and Ly-6C distinguish the two main MDSC subtypes, granulocytic (Gr-MDSCs) and monocytic (Mo-MDSCs) ones, respectively (2). Because the Gr-1 antigen is not expressed on human Gr-MDSCs, other markers have been employed. Today, there is no unequivocal immunoprofiling to identify human MDSCs in cancer patients. Different combinations of antigens have been used including CD33, CD11b, CD14, CD15, CD66b, Lin, and HLADR (3, 4). Nevertheless, human Mo-MDSCs are mostly identified as CD14+ cells with negative or low expression of HLADR. Mo-MDSCs express also high levels of CD11b and CD33 antigens. Human Gr-MDSCs are usually defined as CD66b+CD11b+CD15+HLADR- cells and display an intermediate expression of CD33 and a variable expression of CD11b, depending on their maturation stage (1, 5). A third subtype of MDSCs includes the CD34+ fraction (CD11b+CD33+CD14-HLADR-CD34+ cells) defined as immature myeloid cells (IMCs) (6, 7).

Myeloid derived suppressor cells are myeloid cells characterized by their immature state and, most importantly, by their ability to suppress immune system, especially T cell proliferation and activity (8). MDSCs could suppress proliferation and T cell immunological function in patients with different cancers (9–13). Gr-MDSCs and Mo-MDSCs can inhibit CD4+ effector T cells through different mechanisms (2, 14). MDSCs inhibit immune system by multiple mechanism, mostly through inhibition of T cell activation and expansion. Among others, the first identified were the upregulation of nitric oxide synthase 2 (NOS2), reactive oxygen species (ROS), and overexpression of arginase 1 (15, 16). The upregulation of NOS2 and arginase1 leads to a deficiency of l-arginine, an amino acid indispensable for function and proliferation of T lymphocytes and for CD3ζ-chain expression of the T cell receptor (17, 18). Furthermore, NOS2 activity generates nitric oxide (NO), ROS, and peroxynitrate. Therefore, its upregulation leads to an accumulation of NO that suppresses T cell activity through the inhibition of IL-2 pathway (19). In addition, the increased peroxynitrate production results in the nitration of the CD8 TCR with inhibition of CD8+ T cell activity (20).

More recently, several other mechanisms were identified, including upregulation of cyclooxigenase-2 and prostaglandin E2 (21), induction of regulatory T cells (22–24), production of TGF-β (25), depletion of cystein (26), and downregulation of T cell l-selectin expression (27). Also, inhibition of NK function by MDSCs via downregulation of the activating receptor NKG2D has been reported (1, 28). The specific mechanisms used by MDSCs are microenvironment-dependent.

Despite decreased plasma arginine concentrations have been observed in patients with an immunosuppressive state, some authors have failed to show any detectable benefits of l-arginine supplementation (29). As MDSCs utilize different mechanisms to immunosuppress innate and adaptive anti-tumor immunity, targeting directly MDSCs might be a better approach to overcome MDSC-dependent immune dysfunction.

This review summarizes the current knowledge for human MDSCs and their immunosuppressive function in chronic myeloid leukemia (CML). We will also provide a critical comparison of Gr-MDSCs and PMNs showing a strong potential immune escape mechanism in CML patients created by myeloid cells (6). Finally, we will discuss potential application of MDSCs for the monitoring of MDSCs in patients who have discontinued TKIs therapy, in order to evaluate if their levels could correlate with disease relapse (6).

## CML and Immune Dysfunction

Chronic myeloid leukemia is characterized by the reciprocal chromosomal translocation t(9;22)(q34;q11), leading to the formation of the Philadelphia chromosome. This encodes the constitutively active Bcr-Abl tyrosine kinase, which profoundly affects proliferation, apoptosis, and cell adhesion signaling pathways (30). The majority of patients are diagnosed in chronic phase (CP), showing an expansion of myeloid lineage cells that are maintained by a small subset of CD34+/CD38 – leukemic stem cells (LSCs) (31). Refractoriness of these LSCs to therapy may result in progression to blast crisis (BC), characterized by differentiation arrest and a disease more akin to that of an acute leukemia. The advent of TKIs has drastically changed the treatment outcome of CML. Imatinib was the first TKI approved, and has been considered the standard of care for more than a decade. Although the therapy with Imatinib is considered a major advance in oncology, a significant group of patients still develop drug resistance (32). Second generation compounds, namely Dasatinib and Nilotinib, are highly effective in those who fail Imatinib as well as in newly diagnosed patients (33).

In CML, like in other malignancies, the immune system is impaired favoring immune escape of the malignant cells (6). CD4+ T cells, central components of effective immune response against tumor, appear to be anergic to the leukemia cells (34, 35), and show low levels of TCR-ζ chain expression compared with T cells of healthy subjects (36). The regulation of immune activation has also been found to be altered in CML. Bachy et al. (37) showed that regulatory T cells (T-reg) are significantly increased in CML patients with intermediate or high-risk Sokal scores compared to the low risk (LR) patients. CML patients also express higher levels of programed death receptor ligand 1 (PD-L1) on myeloid cells (including also CD34+ stem cells), compared to control subject cells, and its receptor PD-1 is expressed on T-cells. By binding to the inhibitory receptor PD-1 on T-cells, PD-L1 is able to suppress the T-cell effector functions (38). The same study showed that the interruption of PD-1/PD-L1 interaction enhanced T cell function.

Already there is a promising evidence indicating that some CML patients can stop Imatinib treatment without suffering disease relapse after achieving a complete molecular response (CMR), although they have a minimal amount of residual leukemia cells left (39, 40). This implies that the immune system is able to restrain the tumor cell expansion, and that the TKI therapy has at least restored the function of the normal immune system or probably in some cases further strengthened it (6). Unfortunately, molecular relapse is observed after cessation of Imatinib in 61–66% of CML patients, previously in CMR (41, 42), presumably due to the reactivation of dormant CML LSCs that are resistant to TKI-induced leukemic cell ablation. Thus, current research efforts aim to develop additional therapies to target these TKI-refractory CML LSCs.

## MDSCs Expansion in CML

As in a variety of cancers, MDSCs play a central role in anti-tumor immune response in hematological malignances (7, 43–48), but little is reported on the role of MDSCs in CML. Christiansson et al. investigated for the presence of MDSCs and Arg-1 in CML patients (38). Since Sokal high risk (HR) patients have an increased risk of relapse after TKI treatment cessation (41), the purpose of the authors was to understand the immune status of Sokal HR and LR CML patients. The study showed an increase of the Gr-MDSCs subset (defined as CD11b+CD14-CD33+) that was limited to HR patients. In fact, in their cohort of CML, MDSC levels appeared similar in the CML patient group compared to control individuals. Dividing patients into HR and LR groups, the HR patients showed statistically significant higher MDSC levels both in respect to LR group and control individuals. Also, the expression of arginase 1 as well as its plasma levels were increased in the patients compared to healthy controls. The same authors showed that CD34+ cells (mostly tumor cells) of CML patients expressed MDSC markers; however, MDSCs were identified on both CD34- and CD34+ cell populations. The effect of TKIs on MDSCs and arginase 1 was not evaluated in the Christiansson’s study. However, if the authors demonstrated higher levels of MDSCs in HR patients compared to LR ones, the correlation with worse prognosis for CML needs to be confirmed in a larger study. Furthermore, functional suppressive assays, essential for the evaluation of MDSCs, due to the lack of a unique surface markers signature for their identification (49) were also missing in this study. In addition, it should be underlined that in the Christiansson’s study, the evaluation of MDSC phenotype was conducted in cryopreserved samples, and some studies have shown that separation of Gr-MDSCs from frozen samples leads to a significant decrease in MDSCs viability and function (6, 50).

**Figure 1:**
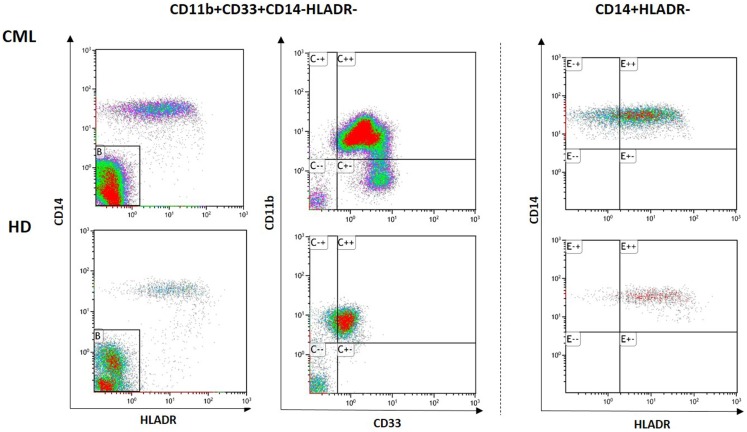
**Representative data of circulating Gr-MDSCs and Mo-MDSCs cells in PB from one healthy donor (HD) and one CML patient at diagnosis**. Flow cytometry analysis was performed with gates set on either CD11b+CD33+CD14-HLADR- or CD14+HLADR- cells populations.

Recently, we have analyzed both Gr-MDSCs and Mo-MDSCs subsets in fresh samples of CML patients at diagnosis and following them during treatment with Imatinib (6). Representative flow cytometry analysis for one patient and one healthy donor is shown in Figure 1. Analyzing by flow cytometry the percentage of MDSCs cells identified as CD11b+CD33+CD14-HLADR-(Gr-MDSCs) and CD14+HLADR-(Mo-MDSCs), we discovered that both subpopulations were significantly increased in patients at diagnosis compared to healthy subjects and decreased to normal levels after Imatinib therapy. No correlation was observed between the percentage of MDSCs and age, nor with leukocytosis, Sokal risk, or the response to TKI therapy. Our set comprised only three patients who were resistant to Imatinib; they showed very high percentages of MDSCs, although not the highest observed. Using magnetic separation, Gr-MDSCs and Mo-MDSCs were analyzed for BCR/ABL expression by real-time polymerase chain reaction (PCR). Both subpopulations showed the oncoprotein expression confirming that they are part of the tumor clone. The series of our study included few patients belonging to HR group, and therefore we could not verify if MDSC levels correlate with Sokal risk.

Expression of arginase 1 and its circulating levels in the serum resulted higher in patients at diagnosis in respect to healthy controls and decreased during Imatinib treatment. Furthermore, the percentage of Gr-MDSCs correlated with arginase 1 protein levels in the serum of patients at diagnosis.

## MDSCs Promote the Expansion of T-reg

**Figure 2:**
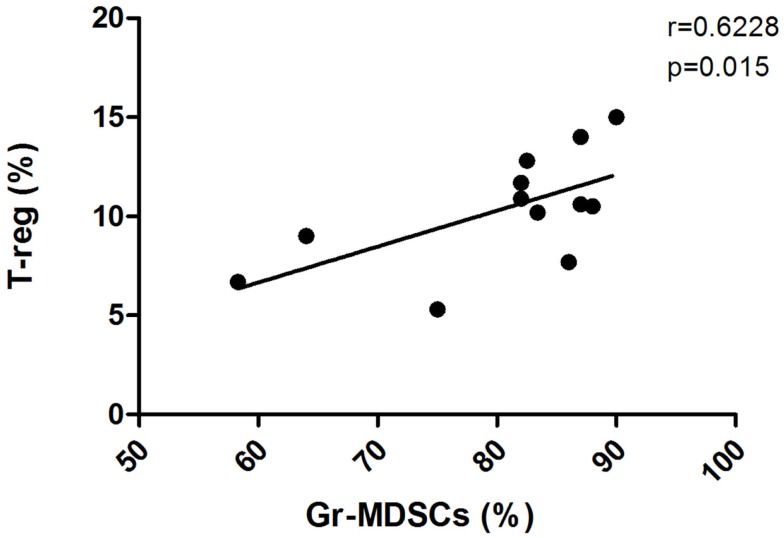
**Frequency of circulating T-reg cells in PB was calculated by cytometric analysis with gates set on CD4+ cells, and the results presented as the percentage of CD25+Foxp3+cells in CD4+ cells**. The figure shows the correlation of the percentages of Gr-MDSCs cells and T-reg in PB from CML patients at diagnosis.

Immunosuppressive CD4+CD25hiFoxP3+ T-reg cells play a central role in the induction of tolerance to cancer antigens. They include both thymus-derived natural T-reg (nT-reg) and locally induced T-reg (iT-reg) cells. Both subpopulations employ contact-dependent and contact-independent mechanisms to constrain the activation of effector T cells (51, 52). Naive CD4+CD25- T cells can be converted into iT-reg cells after exposure to antigen in the presence of cytokines such as TGF-β or IL-10 (53, 54). It has been demonstrated that the administration of progenipoietin-1 (a synthetic granulocyte colony-stimulating factor/Flt-3 ligand molecule) to donors in an allogeneic bone marrow transplantation model generated MDSCs, which upon transfer suppressed the initiation of graft-versus-host disease (GVHD) in recipients by inducing a population of MHC class II-restricted, interleukin 10 (IL-10)-producing T-reg (55). Similarly, in a colon carcinoma murine model, MDSCs either generated or expanded the pool of CD4+CD25+FOXP3+ T-reg (22). Moreover, using the A20 B-cell lymphoma model, Serafini and colleagues showed that the expansion of a preexisting pool of T-reg can be mediated by MDSCs (23). They described MDSCs as tolerogenic antigen presenting cells with ability in antigen uptake and presentation to tumor-specific T-reg. MDSC-mediated T-reg induction required arginase, but was TGF-β independent. Inhibition of MDSC function abrogated T-reg proliferation and tumor-induced tolerance. Many authors provide evidence of an increased frequency of T-reg cells in CML patients at diagnosis in association with higher Sokal scores and higher levels of BCR-ABL transcripts, indicating that an immune response may be important in the control of CML (37, 56). In addition, T-reg number was significantly lower in patients with CP CML versus the accelerated and blast phases, and was significantly lower in patients with CMR compared to those patients without CMR (57). To understand if there is a correlation in CML patients between MDSCs and T-reg frequency, we investigated the levels of both circulating lymphoid and myeloid subpopulations founding a direct correlation between the percentages of T-reg and Gr-MDSCs at diagnosis (Figure 2) (6). The percentage of T-reg cells was higher in patients at diagnosis compared to healthy donors and decreased to the normal levels after Imatinib treatment, confirming the correlation with the levels of Gr-MDSCs.

## Gr-MDSCs and Mature Neutrophils Display Immunosuppressive Properties in CML Patients

Neutrophils take action to innate and adaptive immunity, but their role in cancer has been unclear. Munder et al. demonstrated that arginase activity is specific of human circulating polymorphonuclear cells (PMNs) (58). The enzyme localizes to the azurophil granules and, once released, specifically depletes extracellular arginine inducing profound suppression of various T cell functions (59). Activated PMNs producing arginase1 has been found in the peripheral blood and placenta of pregnant women, suggesting that arginase 1 is important in suppressing the maternal immune responses against the fetus (60). Granulocyte activation and degranulation have been found in abscesses of patients where pus (which contains a large number of activated PMNs) suppresses T cell proliferation and activity (58). Therefore, although PMNs are important in controlling acute infections, they act inhibiting T cell activity in a chronic inflammatory microenvironment where malignant cells are not eliminated. Since arginase 1 has a central role in T cell suppression in both Gr-MDSCs and PMNs in several cancers (61–63), especially in CML characterized by an expansion of granulocytes, it is important to compare Gr-MDSCs with autologous PMNs. First, we demonstrated a significant overlap between Gr-MDSCs and PMNs using the CML as model. As CML Gr-MDSCs, CML PMNs were able to significantly suppress T cell proliferation; no inhibition was observed after incubation with Gr-MDSCs and PMNs from healthy donors (6). Both arginase 1 protein and its enzymatic activity were higher in CML PMNs than autologous Gr-MDSCs, demonstrating a critical role of CML PMNs in the tumor microenvironment. All functional experiments were performed using fresh blood.

Even if Gr-MDSCs and PMNs are phenotypically and functionally different myeloid subsets, they share many common features (6). First, Gr-MDSCs have similar functions to PMNs such as immunosuppression, angiogenesis (64, 65), invasion, and metastasis (66, 67). As PMNs (63), MDSCs acquire strong immunosuppressive activity after activation (68). Furthermore, human Gr-MDSCs are identified by a set of antigens (CD11b, CD14, CD15, CD33, CD66b, CD16, and HLADR), which are well-established markers for PMNs (3, 4). Therefore, the significant overlap between Gr-MDSCs and PMNs concerns both their activity and their immunophenotype. Human MDSCs from renal cell carcinoma (RCC) patients has been described as a subpopulation of activated PMN cells expressing markers of mature activated granulocytes, including high levels of CD66b and CD11b and low levels of CD62L and CD16 (10, 69). These cells degranulated and released arginase 1, resulting in low levels of l-arginine in plasma. Therefore, in RCC patients, activation of normal PMNs induces phenotypic and functional changes similar to MDSCs. Despite the low percentage of IMCs identified as CD11b+CD33+CD14-HLADR-CD34+ cells, CML Gr-MDSCs are more immature cells compared to autologous PMNs, and showed lower levels of expression of CD11b, CD15, and CD16, and lower Arg1 expression and activity (6).

**Figure 3:**
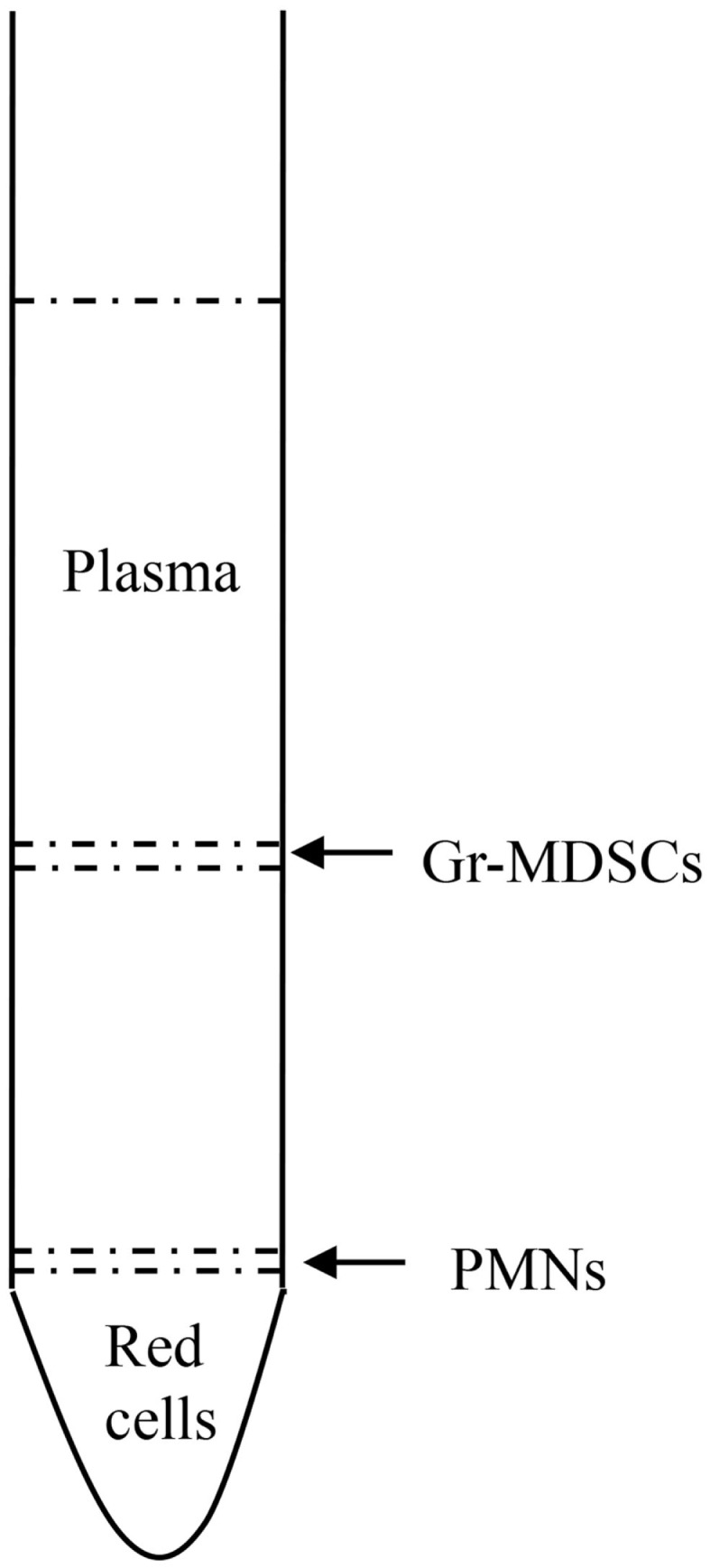
**Isolation of Gr-MDSCs after blood centrifugation over density gradient**. Mature neutrophils typically sediment on top of red cells, whereas mature activated neutrophils and immature granulocytes, as well as Gr-MDSCs, sediment within the mononuclear cell fraction.

In contrast to conventional PMNs collected from the normal density neutrophil fraction on top of red cells, Gr-MDSCs are isolated from the mononuclear cell fraction in density gradient of blood (Figure 3). In fact, Gr-MDSCs are defined as low-density immature cells with neutrophil-like morphology (1, 70). Whether Gr-MDSCs are specialized subsets of neutrophils or originate through an altered process of granulopoiesis is still unclear (3). The recent observations that Mo-MDSCs can differentiate into Gr-MDSCs in tumor-bearing mice and in patients with multiple myeloma have further complicated the scenario (71). Since tumor associated PMNs and Gr-MDSCs seem to represent functional states of cells originating from the same cell type (1), in every study it may be important a careful comparison of Gr-MDSCs and PMNs from the same diseased individuals.

## Concluding Remarks

Among immunosuppressive mechanisms, MDSCs play a central role in anti-tumor immune response in hematological malignancies including CML.

Therapy with TKIs has changed the course of the CML, but these drugs are not able to eradicate CML clone (72, 73). Rohon et al. (74) evaluated the immunophenotype profiling of lymphocytes from CML patients during therapy with Imatinib, and found that the immunoprofile resembled healthy subjects, while Mustjoki et al. (75) showed that treatment with dasatinib resulted in a favorable outcome in Ph+ leukemia with clonal expansion of NK or T cells. The recent study by Mahon et al. (41) demonstrated that approximately 60% of CML patients who stopped therapy with Imatinib after a period of durable CMR relapsed molecularly within the next 6 months. Another study reported that patients who had maintained a stable CMR for at least 2 years with Imatinib therapy stopped therapy, but DNA PCR found CML cells again (76). Therefore, in these patients, the immune system is important in maintaining complete remission. In addition, it has been found that NK are important in controlling the leukemic cell growth; in fact, increased levels of NK cell seem to correlate with the successful Imatinib cessation (77). All these observations, together with the finding of BCR/ABL transcripts in some healthy subjects (78), support the idea that in some patients the immunity could exert an immune surveillance against cancer cells, while an inhibition of this control may lead to a permissive environment for development and progression of leukemia. In this scenario, we hypothesize that Gr-MDSC could play a significant role in favoring the development of CML and its progression. Gr-MDSCs are in fact increased in all CML patients at diagnosis and their level decrease after an effective treatment. We have demonstrated that, in CML, Gr-MDSCs are characterized by a high production of Arginase and it, likely that, is through this protein that they are able to suppress the activity of T lymphocytes. In fact, Gr-MDSCs from CML patients are able to suppress normal lymphocytes activation with a dose-dependent activity. In addition, the suppressive cell population known as T-reg is increased in CML patients at diagnosis and is directly correlated with the amount of MDSC. We have also demonstrated that in CML, the cells that have a MDSC phenotype harbor the genetic defect characteristic of CML, and therefore they at least in part belong to the neoplastic clone. This observation further underlines the lack of knowledge on the mechanisms of MDSC development in CML and other hematological neoplasms. Do these cells derive from myeloid cells (normal or neoplastic) that are induced to become immunosuppressive by cytokines produced by tumors? Is this a special immunosuppressive cell population directly produced by bone marrow in response to a neoplastic stimulus?

In addition, it seems clear that there is a significant overlapping between MDSC and neutrophils and, in our studies, we have found that even mature granulocytes from CML may exert a strong immunosuppressive action on T lymphocytes.

Future studies will be oriented at evaluating the reasons for the development of MDSC, the identification of the cytokines involved in this process, and the possible mediation of other population such as the mesenchymal cells in the induction of the MDSC phenotype. Further study will be also conducted in order to clarify the immunological features of CML patients who relapse after Imatinib discontinuation. In this context, the monitoring of MDSCs in CML patients who have discontinued therapy could be of interest in order to evaluate if their increase could correlate with the restarting of the leukemic growth.

## Conflict of Interest Statement

The authors declare that the research was conducted in the absence of any commercial or financial relationships that could be construed as a potential conflict of interest.
